# The Effect of Silver Nanoparticles on Antioxidant/Pro-Oxidant Balance in a Murine Model

**DOI:** 10.3390/ijms21041233

**Published:** 2020-02-12

**Authors:** Anca Oana Docea, Daniela Calina, Ana Maria Buga, Ovidiu Zlatian, M.M.B. Paoliello, George Dan Mogosanu, Costin Teodor Streba, Elena Leocadia Popescu, Alexandra Elena Stoica, Alexandra Catalina Bîrcă, Bogdan Ștefan Vasile, Alexandru Mihai Grumezescu, Laurentiu Mogoanta

**Affiliations:** 1 Department of Toxicology, University of Medicine and Pharmacy of Craiova, 200349 Craiova, Romania; 2 Department of Clinical Pharmacy, University of Medicine and Pharmacy of Craiova, 200349 Craiova, Romania; 3 Department of Biochemistry, University of Medicine and Pharmacy of Craiova, 200349 Craiova, Romania; anamaria.buga@umfcv.ro; 4 Department of Microbiology, University of Medicine and Pharmacy of Craiova, 200349 Craiova, Romania; ovidiu.zlatian@gmail.com; 5 Graduate Program in Public Health, Center of Health Sciences, State University of Londrina, 60 Robert Koch Avenue, Londrina 86038-350, Brazil; monibas2@gmail.com; 6 Department of Molecular Pharmacology, Albert Einstein College of Medicine, Forchheimer 209,1300 Morris Park Avenue, Bronx, NY 10461, USA; 7 Department of Pharmacognosy and Phytotherapy, Faculty of Pharmacy University of Medicine and Pharmacy of Craiova, 200349 Craiova, Romania; mogosanu2006@yahoo.com; 8 Department of Research Methodology, University of Medicine and Pharmacy of Craiova, 200349 Craiova, Romania; costinstreba@gmail.com; 9 Doctoral School University of Medicine and Pharmacy of Craiova, 200349 Craiova, Romania; popescu.elena88@yahoo.com; 10 Department of Science and Engineering of Oxide Materials and Nanomaterials, Faculty of Applied Chemistry and Materials Science, Politehnica University of Bucharest, 011061 Bucharest, Romania; elena_oprea_93@yahoo.co.uk (A.E.S.); ada_birca@yahoo.com (A.C.B.); grumezescu@yahoo.com (A.M.G.); 11 Department of Histology, University of Medicine and Pharmacy of Craiova, 200349 Craiova, Romania; laurentiu_mogoanta@yahoo.com

**Keywords:** silver nanoparticles, oxidative stress, antioxidant activity, subacute toxicity

## Abstract

This study aimed to evaluate the subacute effect of two types of Ag-NPs(EG-AgNPs and PVP-EG-AgNPs) on antioxidant/pro-oxidant balance in rats. Seventy Wistar rats (35 males and 35 females) were divided in 7 groups and intraperitoneally exposed for 28 days to 0, 1, 2 and 4 mg/kg bw/day EG-Ag-NPs and 1, 2 and 4 mg/kg bw/day PVP- EG-Ag-NPs. After 28 days, the blood was collected, and the total antioxidant capacity (TAC), thiobarbituric reactive species (TBARS),protein carbonyl (PROTC) levels, reduced glutathione (GSH) levels and catalase (CAT) activity were determined. EG-Ag-NPs determined protective antioxidant effects in a dose-dependent manner. The exposure to the 4 mg/kg bw/day EG-Ag-NPs determines both in males and females a significant increase in TAC and CAT and a significant decrease in TBARS and PROTC only in females. The PVP-EG-AgNPs showed a different trend compared to EG-AgNPs. At 4 mg/kg bw/day the PVP-EG-AgNPs induce increased PROTC levels and decreased GSH (males and females) and TAC levels (males). The different mechanisms of EG-AgNPs and PVP-EG-AgNPs on antioxidant-/pro-oxidant balance can be explained by the influence of coating agent used for the preparation of the nanoparticles in the formation and composition of protein corona that influence their pathophysiology in the organism.

## 1. Introduction

Nanoparticles (NPs) have multiple applications in biomedicine, such as controlled drug administration and diagnostic applications [1,2,3,4,5,6,7]. Silver nanoparticles (AgNPs) showed many beneficial effects mainly due to their antibacterial effects, both gram-negative and gram-positive, being effective also against strains with a high degree of virulence [8,9,10,11,12]. With the development of nanotechnology and the use of nanoparticles especially in the medical and pharmaceutical fields, the need for investigation of their toxic effects is essential. Nanoparticles used in the pharmaceutical field have several advantages in improving the targeted delivery of drugs and decreasing their toxicity. Furthermore, metal nanoparticles can be combined with infrared light, radio waves or magnetic field and used for thermal ablation of diseased tissues [13,14,15].

Taking into consideration all the advantages of nanoparticles, evaluation of their toxicity is critical. In order to approve a nano-formulation, a complete pharmacological and toxicological profile is essential [16,17,18].

Recently, several silver nano-formulations have been designed and tested for their potential pharmacological effects as potent antibacterial drugs [7,19,20,21] and promising antitumor agents [22,23]. These nano-formulations differ in size, shape, and surface coating [24,25,26].

Until now, several studies investigated the antibacterial mechanism of action of Ag-NPs. This mechanism is influenced by several factors as NPs diameter, shape, surface changes. Sharma etal. have shown that the antibacterial effect of Ag-NPs is produced in a dose-dependent manner, mainly against gram-negative bacteria, and this antibacterial effect is independent of the acquirement germ resistance to antibiotics. The main mechanisms by which the silver nanoparticles showed their antibacterial properties were by fixation and penetration of the cell wall and modulation of cell signaling [27]. Pal et al. demonstrated that silver nanoparticles interact differently, depending on the shape, with *Escherichia coli*. Thus, truncated triangular silver nanoparticles had the highest biocidal activity, compared to spherical and rod nanoparticles and ionic silver [28]. Morones et al. [29] used different types of gram-negative bacteria to test the antibacterial activities of silver nanoparticles in the 1–100 nm range. Antibacterial activity of Ag-NPs against gram-negative bacteria has been shown to be divided into three steps: (1) nanoparticles with dimensions of 1–10 nm are capable todrastically disrupt normal functions of bacteria, such as permeability and respiration by attaching to the surface of the cell membrane; (2) these nanoparticles are capable of penetrating into the bacterium and causing further damage through possible interaction with compounds containing sulfur and phosphorus, such as DNA; (3) the nanoparticles release silver ions, which will further contribute to the bactericidal effect of the silver nanoparticles [29]. Smekalova et al. reported that the antibacterial activity of silver nanoparticles also depends on surface changes (surfactants/polymers) [30]. As a summarization, the three best-known antibacterial mechanisms of AgNPs are (1) silver ion uptakeby the bacterial cell, followed by disruption of ATP production and DNA replication, (2) generation of reactive oxygen species (ROS) by silvernanoparticles and silver ionsand (3) direct damage of cell membranes by silver nanoparticles. However, further investigations are needed to clarify these mechanisms, especially the issue of the affinity of silver nanoparticles for bacterial proteins containing sulfur and phosphorus and the effects of this affinity on bacterial protein functions [31].

Regarding the use of Ag-NPs as promising antitumor agents, studies have been shown that these effects are correlated with the induction of oxidative and nitro-oxidative stress in cancer cells that lead to mitochondrial disruption and cancer cell death [32].

Their physical and chemical properties also influence their toxicological profile [25]. Route of administration, dose and exposure are critical factors that affectthe degree of toxicity produced by a particular type of NP. In the case of soluble nanoparticles, their toxicity is governed by the components, while in the case of insoluble nanoparticles as stable metal oxides, the mechanism is more complex. Silver nanoparticles (Ag-NPs) have been shown to act by inducing oxidative stress that leads to cytotoxic and genotoxic effects through the induction of DNA damage and apoptosis [33,34,35].These effects depend on colloidal stability and the cellular uptake of the NPs that is influenced by the coated agent, NPs diameter and by the doses used, usually above 10 mg/kg bw in rodents.

In the synthesis of Ag-NPs, the coating process helps in enhancing the stability in the solution by decreasing their agglomeration and preventing the cytotoxicity of Ag-NPs against the living cells, being important factors in decreasing the toxicity of Ag-NPs [36]. Several coating methods have been used for the preparation of Ag-NPS as polymerization, sol-gel method, successive ionic layer absorption and reaction (SILAR) method or biomolecule-mediated Ag-NPs organization. As coating agents in the literature, we find two big categories: organic substances and inorganic substances as metals, metal oxides and metal salts [37]. Ag-NPs have been synthesized using as organic capping agents citric acid, polymers, proteins, polysaccharides, surfactants, etc. From polymers polyethylene glycol and polyvinylpyrrolidone (PVP) are mainly used for stabilizing the AgNPs [38]. The coated Ag-NPs act differently on the organism compared to the uncoated Ag-NPs;hence, their toxicity hasto be evaluated independently in vitro and in vivo.

The generation of ROS by Ag-NPs had a double impact on the therapeutic utilization of these NPs as this mechanism is implicated both in therapeutical efficacy and toxicity. In this study, we synthesized two types of Ag-NPs, one functionalized with ethylene glycol (EG-Ag NPs) and the other functionalized with polyvinylpyrolidone and ethylene glycol (PVP-EG-Ag NPs), and we evaluated the subacute (28 days of intraperitoneal administration) effect on antioxidant/pro-oxidant balance in rats as NPs effects on this can influence both the toxicity and the clinical efficacy of Ag-NPs.

## 2. Results

### 2.1. Transmission Electron Microscopy (TEM) and Selected Area (Electron) Diffraction (SAED)

**Figure 1:**
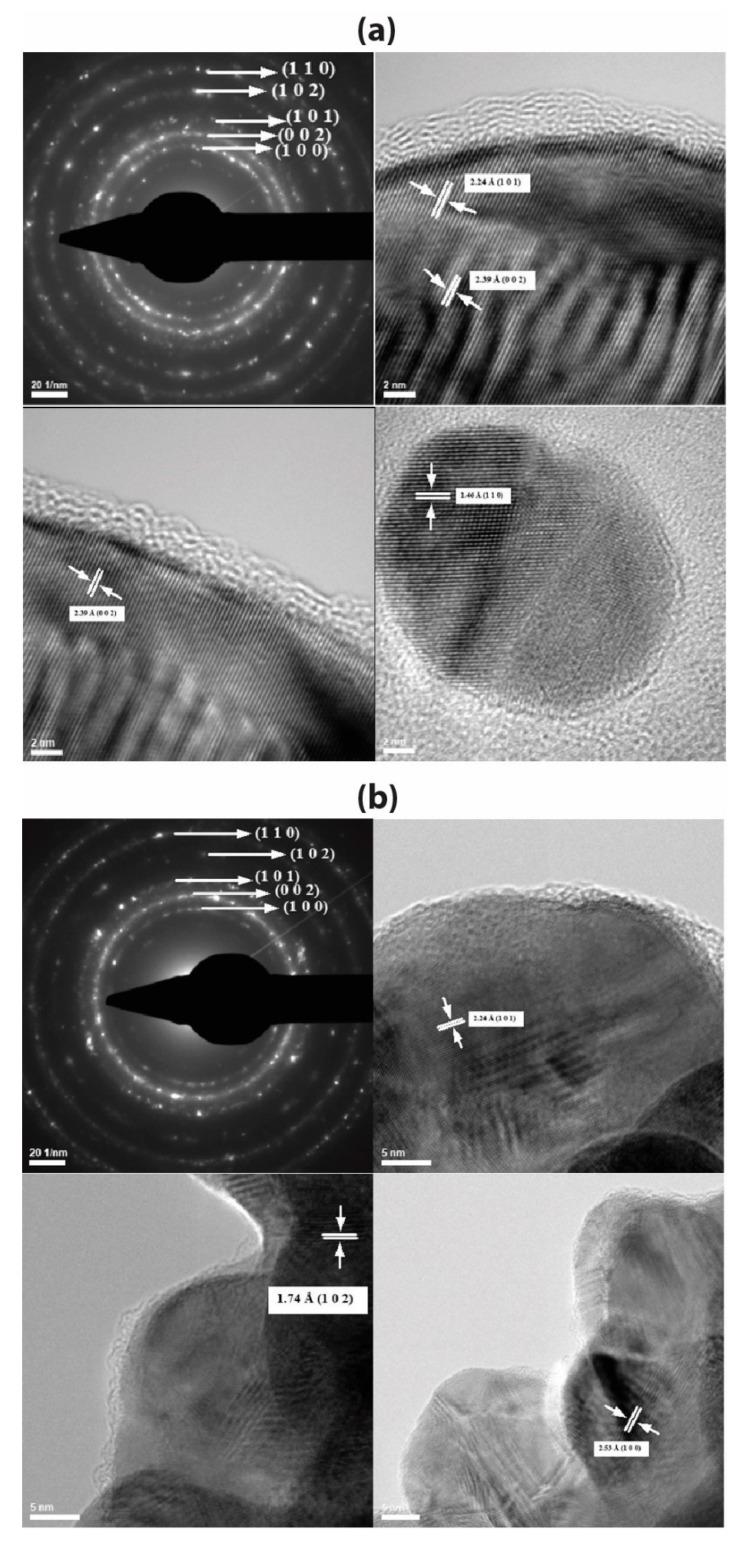
images recorded for (**a**) EG-AgNPs and (**b**) PVP-EG-AgNPs.

**Figure 2:**
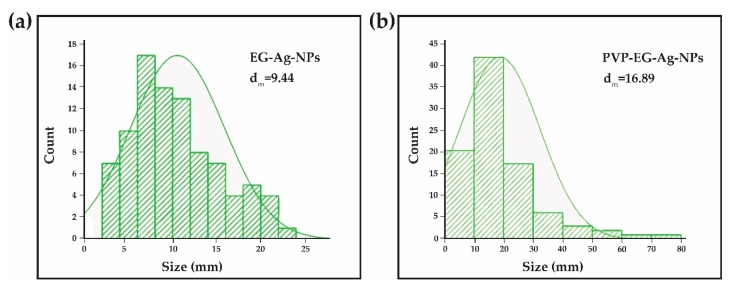
Histogram indicating the particle size distribution: (**a**)Ag-NPs functionalized with ethylene glycol (EG-Ag NPs); (**b**)Ag-NPs functionalized with polyvinylpyrolidone and ethylene glycol.

EG-AgNPs and PVP-EG-AgNPs samples were characterized by Transmission Electron Microscopy; the images obtained are presented in Figure 1. Analyzing the TEM images presented for the two experimental variants, particles of nanometric dimensions can be observed, crystalline, covered by a phase that has low crystallinity that corresponds to the organic components used in the obtaining process (EG and PVP). The EG-AgNPs have an average diameter of 9.44 nm with a Zeta Potential of −14.49 mV, while PVP-EG-AgNPs particles have an average diameter of 16.89 nm (Figure 2) with a Zeta Potential of −47.94 mV, and the coating has thicknesses of 1–3 nm.

The SAED pattern provides information about the crystallinity of the characterized samples (EG-AgNPs and PVP-EG-AgNPs). In the case of both experimental variants, it is observed, from the analysis of the diffraction rings (identified and measured), that the only crystalline phase present is the hexagonal silver. It has been identified and corresponds to the ICDD file [PDF card no. 01-071-5025].

### 2.2. The Effect of EG-AgNPs on Anti-Oxidant Markers

#### 2.2.1. TAC Levels

TAC levels increased after exposure to EG-AgNPs in all the treated groups compared to the control group both in males and females. The increase was not dose-dependent. The females treated with 2 mg/kg bw showed a higher increase expressed as a percentage compared to the control (135.3%), followed by female rats treated with 1 mg/kg bw (122.8%) and the 4 mg/kg bw (11.6%), as shown in Table 1 (*p* < 0.05). The same trend was also observedin male rats, in 1 mg/kg bw group, the TAC levels increased compared to the control group with 106.2%(*p* < 0.05), while in 2 mg/kg bw and 4 mg/kg bw groups the increase was with 55.2%(*p* < 0.05)and with 5.7%(*p* > 0.05), respectively (Table 1).

#### 2.2.2. GSH Levels

Regarding GSH levels, the trend was slightly different in males compared with females. In males, a decrease of GSH levels was observed in 1 mg/kg bw group compared to control and then a dose-dependent increase in 2 and 4 mg/kg bw groups without reaching statistical significance. In females, an increase of GSH levels was observed first compared to control in 1 mg/kg bw group, without reaching the statistical significance, then a decrease compared to control in 2 mg/kg bw group (*p* < 0.05) followed by a slight increase compared to control in 4 mg/kg bw group (*p* > 0.05) (Table 1).

#### 2.2.3. CAT Activity Levels

CAT levels decreased in the 1 mg/kg bw group treated males compared to control and then increased in a dose depending manner in 2 and 4 mg/kg bw group, but without reaching the statistical significance. In females, we observed a dose-dependent increase of CAT levels compared to control in 1, 2 and 4 mg/kg bw group, respectively, reaching the statistical significance only in 4 mg/kg bw group (Table 1).

### 2.3. The Effect of PVP-EG-AgNPs on Anti-Oxidant Markers

#### 2.3.1. TAC Levels

Exposure to PVP-EG-AgNPs showed different effects on TAC levels in male compared to female rats. In male rats exposed to 1 mg/kg bw PVP-EG-AgNPs, increased TAC levels were noted compared to the control (43.4%)(*p* < 0.05), while treatment with 2 mg/kg bw or 4 mg/kg bw PVP-EG-AgNPs led to decreased TAC levels compared with the controls (5.1 and 15.9%, respectively), reaching statistical significance only for 4 mg/kg bw group(Table 2). In female rats the nanoparticle treatment led to 73.1% increase in TAC levels compared to controls after exposure to 1 mg/kg bw PVP-EG-AgNPs (*p* < 0.05), followed by a decrease after exposure to 2 mg/kg bw or 4 mg/kg bw PVP-EG-AgNPs compared to the control, but without reaching the statistical significance (*p* > 0.05) (Table 2).

#### 2.3.2. GSH Levels

The nanoparticle treatment influences the GSH levels in a different manner between males and females. In males, the exposure to 1 and 2 mg/kg bw PVP-EG-AgNPs determines an increased compared to controlwithout reaching the statistical significance (*p* > 0.05), while theexposure to 4 mg/kg bw PVP-EG-AgNPs decreased the GSH levels compared to control(*p* < 0.05) (Table 2). In females, the nanoparticle treatment determined a dose-dependent decrease in GSH levels compared to control after treatment with 1, 2 and 4 mg/kg bwPVP-EG-AgNPs respectively, reaching the statistical significance only for 2 and 4 mg/kg bwgroups (*p* < 0.05)(Table 2).

#### 2.3.3. CAT Activity Levels

The effects of PVP-EG-AgNPs administration on CAT levels determine in males from 1 mg/kg bw group a decrease compared to control followed by an increase compared to control in 2 and 4 mg/kg bw groupsPVP-EG-AgNPsreaching the statistical significance only for 2 mg/kg bw group (Table 2). In females, the treatment with PVP-EG-AgNPs led to an increased compared to the control in all the treated groups, reaching the statistical significance only for 1 mg/kg bwgroup (*p* < 0.05) (Table 2).

### 2.4. The Effect of EG-AgNPs on Pro-Oxidant Markers

#### 2.4.1. TBARS Levels

The TBARS levels increased both in malesand females after treatment with 1 mg/kg bw EG AgNPs compared to control with 77.65% and 1.22%, respectively, reaching the statistical significance only for males (Table 3). Exposure to 2 mg/kg bw EG AgNPs and 4 mg/kg bw EG AgNPs led to decreased TBARS levels compared to controls both in males and in females, but the statistical significance is reached only for females (Table 3).

#### 2.4.2. PROTC Levels

Treatment with EG-AgNPs determined in male rats increased PROTC levels compared to controls in 1 mg/kg bw group and 2 mg/kg bw group and then a decrease compared to control in 4 mg/kg bw groupwithout reaching the statistical significance (Table 3). In female rats, it was observed an increase in PROTC levels compared to control in 1 mg/kg bw group (*p* < 0.05) and then a decrease compared to control in 2 mg/kg bw and 4 mg/kg bw groups, reaching the statistical significance only for 4 mg/kg bw group (Table 3).

### 2.5. The Effects of PVP-EG-AgNPs on Pro-Oxidant Markers

#### 2.5.1. TBARS Levels

The exposure to PVP-EG-AgNPs led to a dose-dependent decrease in TBARS levels compared to control group in males, reaching statistical significance only in 2 and 4 mg/kg bw groups, while in females the highest decrease was reached by the 1 mg/kg bw groups, followed by 2 mg/kg bwthat reached the statistical significanceand 4 mg/kg bw group (*p* < 0.05), respectively (Table 4).

#### 2.5.2. PROTC Levels

In males, treatment with PVP-EG-AgNPs led to decreased PROTC levels compared to controls at 1 (*p* > 0.05) and 2 mg/kg bw (*p* < 0.05), respectively, and then an increase compared to control in 4 mg/kg bw group (*p* < 0.05) (Table 4). In females was observed a decrease compared to control after exposure to 1 mg/kg bw PVP-EG-AgNPs, without reaching a statistical significanceand then an increase in the 2 and 4 mg/kg bw PVP-EG-AgNPs (*p* < 0.05), respectively (Table 4).

## 3. Discussion

The extended use of AgNPs in the pharmaceutical field has raised concernsregarding their health effects. Several studies have investigated the mechanism of toxicity of Ag –NPs suggesting that their cytotoxicity is mainly mediated by induction of reactive oxygen species (ROS). The size of the NPs, their dose and the duration of treatment are critical in mediating their effects [39,40]. Another factor associated with the cytotoxicity of AgNPs is the surface-stabilizing agent [41,42].

In this study, we used two different coatings for the synthesis of nanoparticles: EG and PVP-EG. The aim of this study was to investigate the effect of subacute administration of EG-Ag-NPs and PVP-EG-Ag-NPs (for 28 days) on antioxidant/pro-oxidant balance in rats. The antioxidant/pro-oxidant balance was evaluated by determining the levels of TAC, TBARS, PROTC, GSH and CAT. TAC is the marker that evaluates the antioxidant status of the biological system and is very useful to evaluate the response of the organism against the free radical production [43]. TBARS is one of the oldest and most widely used markers for the evaluation of lipid peroxidation [44]. PROTC is a marker that evaluates the oxidation of the proteins that increase due to oxidative stress [45]. Reduced GSH is considered to be the first line of non-enzymatic antioxidant of the defense system that fights against oxidative stress and gets depleted in the oxidative stress models [46]. CAT is the enzyme that converts superoxide to water and molecular oxygen and prevents the formation of hydroxyl radical and other toxic ROS species [47].

We showed that the exposure of rats to EG-Ag-NPs determined protective antioxidant effectswith slight differences between males and females. At doses of 1 mg/kg bw/day, a significant increase in TAC levels associated with a significant increase in TBARS in maleswas observed.In females exposed to 1 mg/kg bw/day, a significant increase in TAC, GSH and PROTC levelswas observed. An explanation for this discrepancy between TBARS level in males and females at the same dose can be explained by the gender-dependent response of the body that affects in a specific way the oxidative damage of the lipids. It is well known that females seem to respond faster than males to oxidative stress displaying increased protection due to estrogen [48,49,50]. However, further investigation should be performed in order to elucidate the mechanism that is involved in a gender-dependent response to lipid peroxidation. At the medium, dose tested, the protective effects of the EG-Ag-NPs are more visible especially in females where we observed a significant increase in TAC levels and a significant decrease of TBARS. The exposure to the 4 mg/kg bw/day EG-Ag-NPs determines protective effects against oxidative stress, especially in females, translated by a significant increase of TAC and CAT and a significant decrease in TBARS and PROTC in females. The same trend was present also in males but without reaching the statistical significance. The difference in the patterns observed in males and females can be explained by the gender differences in circulation and elimination of AgNPs [51]. The protective antioxidant effects observed for EG-AgNPs are in line with the study of Singh et al. (2018) that showed the protective effect of AgNPS against chemical-induced hepatotoxicity in rats by re-establishing the antioxidant levels [7]. Antioxidant effects of AgNPsareone of their beneficial effects, which determined their use in a lot of products for biomedical application [52]. AgNPs synthesized by green nanotechnology showed to have antioxidant activity [53,54]. PatilShriniwas et al. (2017) showed that AgNPs synthesized using terpenes extracted from *Lantana camara* L. showed, at doses of 2 mg/mL, antioxidant activity similar to ascorbic acid [55]. It is also worth investigating if these protective effects against oxidative stress are valid in the real-life exposure scenario where the individual is exposed in a chronic manner to a combination of stimuli that can be additive, synergic or antagonist [56,57]. Several studies showed that the combined exposure at low doses can produce non-monotonic responses [58,59,60], and this is worth investigating also for Ag-NPs as the exposure to these molecules usually appearsin combination to other agents that can influence their effects on biological organisms.

The PVP-EG-AgNPs showed a different trend regarding antioxidant/pro-oxidant balance compared to EG-AgNPs.At higher doses, the NPs induce increased protein oxidation translated by increased PROTC levels and decreased GSH both in males and females and TAC levels in males after exposure to 4 mg/kg bw/day. Patlolla et al. showed that evaluating the acute toxicity of oral administration of AgNPs at doses of 50 and 100 mg/kg bw/day, the ROS induction increased compared to the control group [61]. Foldbjerg et al. showed that PVP-coated AgNPs dramatically increased ROS levels in the human monocytic cell line that led to cell apoptosis and necrosis [62]. The mechanism by which the Ag-NPs induce oxidative stress seems to be associated with the generation of intracellular ROS determined by the Ag+ ions found on the surface of the NPs or released from the NPs [23,62,63]. This is also supported by our findings that showed increased protein oxidation translated by increased PROTC levels. Another possible mechanism of Ag-NPs induced oxidative stress is mediated by the affinity of the NPs to the thiol groups, which lead to a reduction of GSH levels and the inability of this molecule to neutralize the ROS [64]. This effect is also supported by our findings, which showed that exposure to PVP-EG-AgNPs produces a decrease in GSH and TAC levels. These results can contribute to the further investigation of the beneficial effects of synthesized PVP-EG-AgNPs in cancer treatment. Ag-NPs have been shown to induce programmed cell death in several cancer cell lines, and these effects have been correlated with the induction of ROS species [23,32].

The coating of Ag-NPs is used to stabilize the NPs by producing electrostatic and electrosteric repulsions between particles. Several studies showed that coating also protects against the cytotoxicity manifested by Ag-NPs [65]. It was demonstrated that the coating of nanoparticles influences the formation and composition of protein corona in the biological fluid that can further influencethe pathophysiology of the NPs in vitro [18,66]. In our study, we demonstrated that the coating agent affects the effects of Ag-NPs on antioxidant/pro-oxidant balance and can be an essential factor in Ag-NPs toxicityor targeted therapeutic effect of Ag-NPs.

## 4. Materials and Methods

### 4.1. Raw Materials

Silver nitrate (AgNO3), Sodium hydroxide (NaOH), Ethylene glycol (EG) and Polyvinylpyrrolidone (PVP) were purchased from Sigma–Aldrich without further purification. All chemicals were of analytical purity and used with no further purification. Deionized water was used throughout the experiment.

### 4.2. Synthesis of EG-AgNPs and PVP-EG-AgNPs

**Figure 3:**
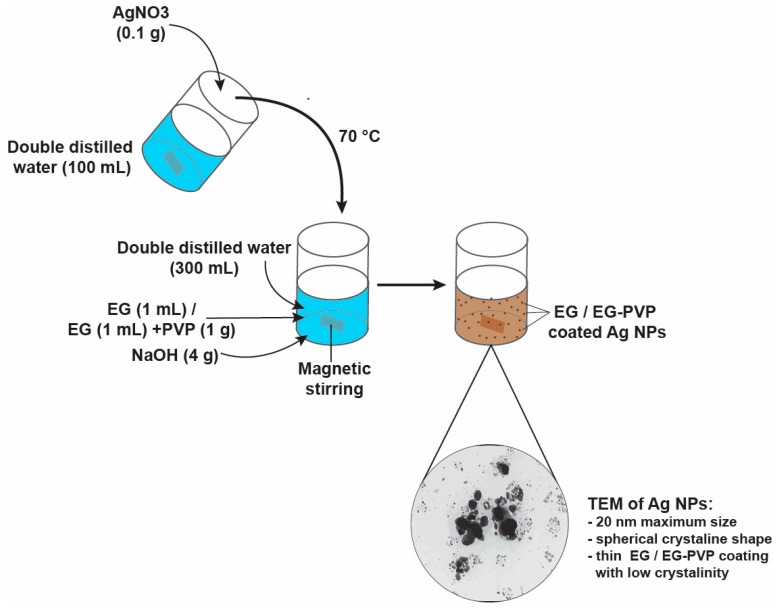
Representation of EG-Ag-NPs synthesis.

The experimental obtentionof silver nanoparticles in the presence of EG and EG/PVP was possible by using a chemical method to reduce the metal precursor, which involved obtaining two solutions necessary for the synthesis process for each particular synthesis. The silver nitrate solutions were obtained by dissolving 1 g of AgNO3 in 300 mL of ultrapure water. The second solution was prepared by dissolving 20 g NaOH and 3 g EG and 1,5 g EG + 1,5 g PVP, respectively, in 400 mL ultrapure water under magnetic stirred at 80°C. Subsequently, silver nitrate solutions were added to the polymer/monomer solutions by dripping and under continuous magnetic stirring. During the process, it was observed the change in the color of the dispersions obtained an aspect correlated with the formation of silver particles. The collection of the synthesized nanoparticles was possible by vacuum filtration of the obtained dispersions. The silver particles were subjected to a triple wash treatment with distilled water and dried at room temperature (see Figure 3).

### 4.3. Characterization of EG-AgNPs and PVP-EG-AgNPs

#### 4.3.1. Transmission Electron Microscopy (TEM)

TEM images were obtained using a high-resolution Tecnai^TM^ G2 F30 S-TWIN transmission microscope equipped with SAED, from the FEI (Oregon, USA). The transmission modewas used at 300 kV, the point and line resolutions were 2 Å and 1 Å, respectively.

#### 4.3.2. Zeta Potential Measurement

Zeta potential measurements were performed using a DelsaMax Pro equipped with a laser at 532 nm. The samples were prepared by dispersing in ultrapure water at room temperature.

### 4.4. Animals

Seventy Wistar rats (35 males and 35 females), 12 weeks old, with a median weight of 343 ± 20 g for males and 236 ± 22 g for females were obtained from the Animal House of the University of Medicine and Pharmacy of Craiova, Craiova, Romania. One week before the start of the study, the animals were acclimatized to the new conditions with constant temperature 22 ± 2°C, humidity between 40% to 60% and dark/light cycle of 12 h/12 h. The animals received food and water ad libitum. The animal experiment was approved by the Ethical Committee of the University of Medicine and Pharmacy of Craiova, Craiova, Romanianumber 89/13.09.2018 and respected all the directives for animal experiments requested by EU Commission Directive 2010/63/EU.

### 4.5. Experimental Design

**Figure 4:**
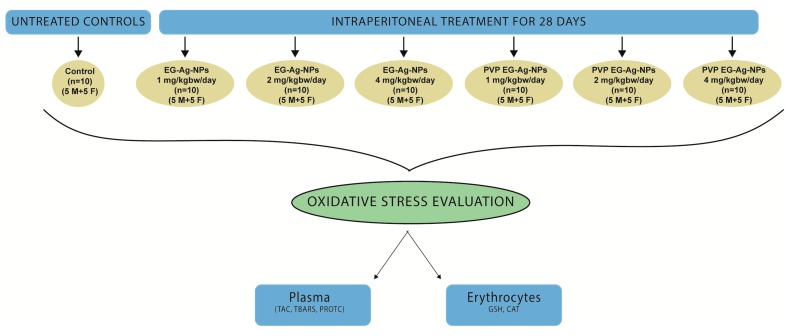
Design of the study.

The rats were assigned to 7 groups (5 males and 5 females per group), one control and 6 treatment groups as depicted in Figure 4. The treatment groups were treated with the dispersion of nanoparticles in ultrapure water at room temperature.

After 28 days of exposure, the rats were immobilized with a restrainer and the blood was collected into ethylenediaminetetraacetic acid (EDTA) vacutainers from tail veins using a 23 G needle.

### 4.6. Oxidative Stress Markers Evaluation

All the reagents for oxidative markers were purchased from Sigma-Aldrich (USA). The blood samples collected in EDTA were centrifuged at 1370× *g* for 10 min at 4 °C, the plasma and erythrocytes were separated and stored at −80 °C for further analysis. The total antioxidant capacity (TAC), thiobarbituric reactive species (TBARS) and protein carbonyl (PROTC) levels were determined in plasma as previously described [58,67,68]. The collected packed erythrocytes were lysed with distilled water (1:1 *v/v*) followed by centrifugation at 4020× *g* at 4 °C for 15 min and then the erythrocyte lysate was collected and used to determine reduced glutathione (GSH) levels and catalase (CAT) activity as previously described [69].The total protein concentration in the plasma was determined by the Bradford assay [70]. A BC-5000 Vet auto hematology analyzer (Mindray, North America) was used to determine the hemoglobin concentration.

Briefly, for TAC analysis, plasma samples were diluted in 0.01 M PBS at neutral pH (dilution factor = 1:25). The diluted samples were mixed with 0.01 M DPPH solution in 1:1 ratio and incubated for half-hour time in a dark chamber at room temperature (RT). After the incubation time, the samples were centrifugated for 3 min at 20,000× *g* at 4°C(Eppendorf 5417 R centrifuge), and the supernatant was collected for spectrophotometric analysis. The samples absorbance at 517 nm (A250) was read using a Hitachi UV-VIS spectrophotometer, and TAC was expressed as mmol of reduced DPPH using the molar extinction coefficient of DPPH (11,500 M^−1^·cm^−1^).

TBARS assay was performed as follows: the 35% TCA was mixed with 0.2 MTris-CL at neutral pH in 1:1 ratio. One part of the plasma sample was added to 9 parts of TCA/TRIS-Cl mixture and incubated at RT for 10 min. After incubation time an equal volume of 2 M sodium sulfate in 0.05 Mthiobarbituric acid (TBA) mixture was added in the sample, heated at 95 °C for 45 min on a water bath and then cooled on ice for 5 min. TCA (70%) was added in the sample and mixed using a vortex. After that, the samples were centrifuged at 15,000× *g* for 3 min (Eppendorf 5417 R centrifuge). The supernatant was collected and analyzed by spectrophotometry at 532 nm. The TBARS level was expressed using the molar extinction coefficient of MDA-TBA abduct (155 mM^−1^ cm^−1^).

PCARB assay was performed as follows: plasma sample and 20% TCA were mixed in 1:1 ratio and incubated on ice for 15 min. After incubation time, the samples were separated by centrifugation at 15,000× *g* for 5 min at 4°C(Eppendorf 5417 R centrifuge), and the supernatant was removed. A volume of 0.5 mL of 0.01 M of DNPH solution was added on the pellet. For the blank, we added 2.5 N HCL. After that, an incubation step was performed for 60 min in a dark chamber with intermittent vortexing. After the incubation step, the samples were centrifuged at 15,000× *g* for 5 min at 4°C(Eppendorf 5417 R centrifuge). After separation by centrifugation, the supernatant was removed, and the pellet was washed three times with ethanol-ethylacetate solution in equal parts (*v/v*). The pellet was then solved in 5 M urea at pH = 2.3 followed by vortex and incubation at 37 °C for 15 min. After incubation time, the samples were centrifuged at 15,000× *g* for 3 min at 4°C(Eppendorf 5417 R centrifuge) and the supernatant was collected and analyzed by spectrophotometry at 375 nm. The PCARB content from total protein concentration (analyzed by the Bradford method) was calculated using the molar extinction coefficient of DNPH (22 mM^–1^ cm ^–1^).

In order to assess the GSH level and CAT activity, we performed the erythrocyte lysate using an equal volume of distillate water, mix by inversion and centrifuged for 15 min at 4020× *g* using a 4°C centrifuge (Eppendorf 5417 R).

GSH level was assessed in erythrocyte lysate as follows: the erythrocyte lysate was treated with 5% TCA (*v/v*), vortex and separated by centrifugation at 28,000× *g* for 5 min at 4°C (Eppendorf 5417 R centrifuge). In the sample, a mixture of 0.01 M DTNB in 0.07 M PBS at alkaline pH (pH = 8) in 1:50 ratio (*v/v*) was added and incubated in a dark chamber at RT for 45 min. After incubation time, the samples were analyzed by spectrophotometry at 412 nm using a UV-VIS spectrophotometer (Hitachi). The GSH concentration was calculated using a GSH standard curve.

For CAT activity analysis, we used 4 microL of 1:10 dilution of erythrocyte lysate sample in 3 ml of 0.07 M PBS at neutral pH. The mixture was incubated at 37 °C for 10 min, and the changes in sample absorbance at 240 nm after peroxide addition wereread using a UV-VIS spectrophotometer (Beckman UV-VIS). The CAT activity was calculated based on the molar extinction coefficient of peroxide and expressed as Unit per mg of hemoglobin (U/mgHb).

### 4.7. Statistical Analysis

All statistical analyses were performed using STATA 13 (StataCorp. 2013. Stata Statistical Software: Release 13. College Station, TX: StataCorp LP). Continuous data were expressed as arithmetic mean (Average) ± standard deviation of the mean. To determine the difference between the groups in normally distributed data, we used a one-way analysis of variance (ANOVA) and Tukey’s post hoc tests. In the case of non-normally distributed data, we used Kruskal–Wallis and post-hoc Mann–Whitney tests with Holm–Sidak adjustment. A value of *p*<0.05 was considered statistically significant.

## 5. Conclusions

EG-AgNPs manifest antioxidant effects that protect against oxidative stress in subacute exposure, while PVP-EG-AgNPs manifest pro-oxidant effects at the same doses and in the same administration regimen. The EG-AgNPs protect against oxidative stress by increasing TAC and CAT levels and decreasing TBARS and PROTC levels. The mechanism of PVP-EG-AgNPs induction of oxidative stress is mediated by the induction of protein oxidation and decreased GSH levels. The different mechanisms of EG-AgNPs and PVP-EG-AgNPs on antioxidant-/pro-oxidant balance can be explained by the influence of the coating agent used for the preparation of the nanoparticles in the formation and composition of protein corona that influence the pathophysiology of the nanoparticles in the organism. Further studies should be carried out in order to evaluate the chronic effects of exposure to these types of NPsand also the beneficial effects of synthesized PVP-EG-AgNPs in cancer treatment based on their properties to induce ROS.

## Figures and Tables

**Table 1:** Markers at 28 days after exposure to different concentrations of EG-AgNPs.

Parameter		Males				Females			
		Control	1 mg/kgbw	2 mg/kgbw	4 mg/kgbw	Control	1 mg/kgbw	2 mg/kgbw	4 mg/kgbw
TAC (mmol DPPH/L)	Average	0.2422	0.4993 *	0.3758 *	0.2561	0.2316	0.5161 *	0.5450 *	0.2584 *
	SD	0.0138	0.492	0.0492	0.0157	0.0159	0.0091	0.0140	0.0060
	% to control		106.2%	55.2%	5.7%		122.8%	135.3%	11.6%
GSH (µmol/g Hb)	Average	1.9408	1.8092	1.9901	2.0559	2.2039	2.4178*	1.9342*	2.2270
	SD	0.1628	0.0601	0.0349	0.0349	0.0233	0.0814	0.0221	0.1506
	% to control		−6.8%	2.5%	5.9%		9.7%	−12.2%	1.1%
CAT (U/g Hb)	Average	177.38	168.5714	185.02	192.96	171.03	171.10	177.78	188.27*
	SD	2.38	28.9076	2.175	1.61	0.56	2.51	1.56	11.25
	% to control		−5.0%	4.3%	8.8%		0.1%	3.9%	10.1%

Notes: * *p*< 0.05 compared to the control group.

**Table 2:** Markers at 28 days after exposure to different concentrations of PVP-EG-AgNPs.

Parameter		Males				Females			
		Control	1 mg/kgbw	2 mg/kgbw	4 mg/kgbw	Control	1 mg/kgbw	2 mg/kgbw	4 mg/kgbw
TAC (mmol DPPH/L)	Average	0.2422	0.3472 *	0.2545	0.2038 *	0.2316	0.4009 *	0.2171	0.2450
	SD	0.0138	0.0016	0.0138	0.0063	0.0159	0.0315	0.0031	0.0039
	% to control		43.4%	−5.1%	−15.9%		73.1%	−6.3%	5.8%
GSH (µmol/g Hb)	Average	1.9408	2.2862	2.1875	0.8224 *	2.2039	2.1382	2.0066 *	1.5461 *
	SD	0.1628	0.1977	0.1047	0.3489	0.0233	0.1163	0.0930	0.0698
	% to control		17.8%	12.7%	−57.6%		−3.0%	−9.0%	−29.9%
CAT (U/g Hb)	Average	177.38	169.84	216.47*	181.84	171.03	199.01*	188.09	179.66
	SD	2.38	5.61	15.43	2.59	0.56	14.73	5.33	13.96
	% to control		−4.3%	22.0%	2.5%		16.4%	10.0%	5.1%

Notes: * *p*< 0.05 compared to control group.

**Table 3:** Markers at 28 days after exposure to different concentrations of EG-AgNPs.

Parameter		Males				Females			
		Control	1 mg/kgbw	2 mg/kgbw	4 mg/kgbw	Control	1 mg/kgbw	2 mg/kgbw	4 mg/kgbw
TBARS (µmol/L)	Average	0.6290	1.1174 *	0.5323	0.4903	0.7032	0.7118	0.4361 *	0.4619 *
	SD	0.0798	0.3934	0.0205	0.0274	0.0274	0.0249	0.0202	0.0195
	% to control		77.7%	−15.4%	−22.1%		1.2%	−38.0%	−34.3%
PROTC (nmol/mg protein)	Average	0.9008	1.0297	0.9450	0.8287	1.0792	1.7134*	0.7076	0.5934*
	SD	0.1029	0.0649	0.1090	0.1711	0.0611	0.7115	0.0257	0.1499
	% to control		14.3%	4.9%	−8.0%		58.8%	−34.4%	−45.0%

Notes: * *p*< 0.05 compared to thecontrol group.

**Table 4:** Markers at 28 days after exposure to different concentrations of PVP-EG-AgNPs.

Parameter		Males				Females			
		Control	1 mg/kgbw	2 mg/kgbw	4 mg/kgbw	Control	1 mg/kgbw	2 mg/kgbw	4 mg/kgbw
TBARS (µmol/L)	Average	0.6290	0.6710	0.4968*	0.5194*	0.7032	0.4677*	0.5065*	0.8129
	SD	0.0798	0.0502	0.0137	0.0068	0.0274	0.0205	0.0205	0.1962
	% to control		−6.7%	−21.0%	−17.4%		−33.5%	−28.0%	15.6%
PROTC (nmol/mg protein)	Average	0.9008	0.6718	2.1341*	3.8725*	1.0792	0.7884	2.5592*	2.5274*
	SD	0.1029	0.0516	0.8640	0.1741	0.0611	0.0320	0.0994	0.4932
	% to control		−25.4%	−136.9%	330.0%		−27.0%	137.1%	134.2%

Notes: * *p*< 0.05 compared to the control group.
